# IL-2/IL-2R Antibody Complex Enhances Treg-Induced Neuroprotection by Dampening TNF-α Inflammation in an In Vitro Stroke Model

**DOI:** 10.1007/s12017-021-08656-0

**Published:** 2021-04-08

**Authors:** Mia C. Borlongan, Chase Kingsbury, Felipe Esparza Salazar, Alma R. Lezama Toledo, German Rivera Monroy, Nadia Sadanandan, Blaise Cozene, Bella Gonzales-Portillo, Madeline Saft, Zhen-Jie Wang, Alexa Moscatello, Jea Y. Lee

**Affiliations:** Department of Neurosurgery and Brain Repair, University of South Florida Morsani College of Medicine, Tampa, FL 33612 USA

**Keywords:** Ischemia, Neurons, Oligodendrocytes, Neural progenitor cells, Inflammation, Immune cells

## Abstract

The present in vitro study showed that IL-2/IL-2R antibody complex facilitates Treg-induced neuroprotection in the oxygen glucose deprivation/reoxygenation (OGD/R) model of stroke. First, we examined the role of IL-2/IL-2R-treated Tregs in OGD/R-exposed rat primary cortical cells (PCCs), which represent the cell type of the ischemic gray matter in the stroke brain. Here, OGD/R induced cell death, which was attenuated by Tregs and more robustly by IL-2/IL-2R-treated Tregs, but not by IL-2/IL-2R treatment alone. Second, we next assessed IL-2/IL-2R effects in OGD/R-exposed human oligodendrocyte progenitor cells (OPCs), which correspond to the white matter injury after stroke. Results revealed that a similar pattern neuroprotection as seen in the gray matter, in that OGD/R triggered cell death, which was ameliorated by Tregs and more effectively by IL-2/IL-2R-treated Tregs, but IL-2/IL-2R treatment alone was not therapeutic. Third, as we begin to understand the mechanism underlying IL-2/IL-2R engagement of Tregs, we investigated the inflammatory response in OGD/R-exposed human neural progenitor cells (NPCs), which recapitulate both ischemic gray and white matter damage in stroke. Similar to PCCs and OPCs, OGD/R produced cell death and was blocked by Tregs and more efficiently by IL-2/IL-2R-treated Tregs, whereas IL-2/IL-2R treatment alone did not alter the ischemic insult. Moreover, the inflammatory marker, TNF-α, was upregulated after OGD/R, dampened by both Tregs and more efficiently by IL-2/IL-2R-treated Tregs but more pronounced in the latter, and not affected by IL-2/IL-2R treatment alone, suggesting IL-2/IL-2R-Treg-mediated modulation of inflammatory response in stroke. Altogether, these observations support the use of IL-2/IL-2R treatment in enhancing the anti-inflammatory effects of Tregs in stroke.

## Introduction

Stroke continues to be one of the most widespread causes of disability and death in adult populations and is projected to amount to more than 200 billion dollars annually in the United States by 2030 (Benjamin et al., 2019). Stroke mainly exists as ischemic or hemorrhagic, although the former comprises 87% of all stroke cases (Tal et al., 2015; Virani et al., 2020). Currently, stroke treatment is limited to tissue plasminogen activator (tPA) and mechanical thrombectomy. tPA treatment has shown to be most effective when administered within 4.5 h of onset (Kim, 2019). However, if tPA is given outside of its therapeutic window, significant risks of hemorrhagic transformation occur (Kim, 2019). Alternatively, mechanical thrombectomy can also be used to treat ischemic stroke; however, it too has limited therapeutic window (6–24 h following stroke) as well as an array of other negative complications (Primiani et al., 2019). Given the current state of tPA and mechanical thrombectomy, novel treatments are needed to expand the therapeutic window.

Transplantation of stem cells, such as bone marrow-derived stem cells (BMSCs), has a potential use as a therapy for this ischemic stroke, as they possess the capacity to restore damaged tissues and to secrete therapeutic substances, including anti-inflammatory factors, altogether abrogating the secondary cell death (DeCarolis et al., 2015; Nguyen et al., 2019). A key cell population of BMSCs, Tregs exert an immunomodulatory function by inducing an anti-inflammatory immune cell phenotype (Suenaga et al., 2015). Tregs play a fundamental role in suppressing the activation of the deleterious immune response and inflammation after stroke (Zarriello et al., 2019).

Shortly after stroke, there is a significant increase in Tregs over several weeks (Hori et al., 2003). However, such endogenous Treg upregulation is not sufficient to halt the progression of the secondary cell death, suggesting that further enhancement and mobilization of Tregs are needed. The IL-2/IL-2R complex selectively expands Tregs (Fontenot et al., 2005). Treatment with IL-2/IL-2R complex reduces stroke-induced inflammation and neurological deficits, coincident with increased Tregs in vivo (Mao et al., 2017).

The concept of IL-2/IL-2R complex-mediated Treg approach represents a novel stroke treatment in that it affords a wider therapeutic window, recapitulates a pharmacologic ligand–receptor interaction, and acts by regulating the inflammation-plagued secondary cell death (Zhang et al., 2018). The present study probed to delineate the direct effects of IL-2/IL-2R complex from Tregs in the in vitro stroke model of oxygen glucose deprivation/reoxygenation (OGD/R) model. To partially capture the impaired neurovascular unit in stroke, we exposed to OGD/R cultured rat primary cortical cells (PCCs), human oligodendrocyte precursor cells (OPCs), and human neural progenitor cells (NPCs), which correspond to gray matter, white matter injury, and combination of both, respectively. Our overarching hypothesis was that if IL-2/IL-2R complex plays a key role in Tregs’ therapeutic effects, then their combined treatment would produce more robust attenuation of OGD/R-exposed cell death compared to stand-alone treatments of IL-2/IL-2R complex or Tregs.

## Methods

### Experimental Design

**Fig. 1:**
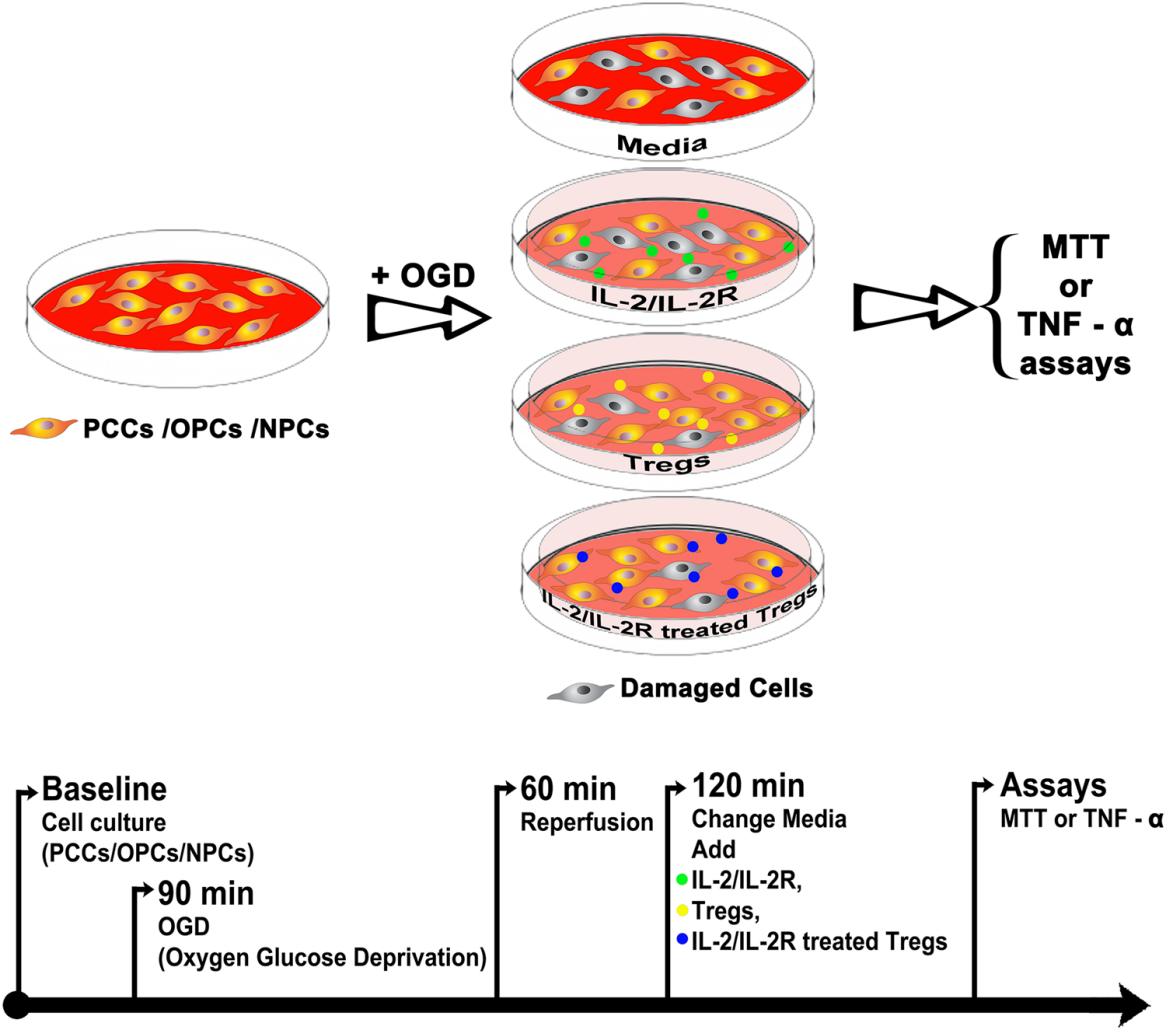
Schematic diagram of experimental design. Targeted cells, including PCCs, OPCs, and NPCs are grown in confluence, then subjected to OGD/R and assigned to one of the following treatment conditions: standard media, IL-2/IL-2R alone (green dots), Tregs alone (yellow dots), or IL-2/IL-2R-treated Tregs (blue dots). The cells are then processed for cell viability (MTT) and inflammation (TNF-α) assays

The experimental study was conducted by investigators blinded to the treatment conditions. The experimental design consisted of exposing the targeted cells (PCCs, OPCs, and NPCs) to oxygen glucose deprivation. The cells were initially exposed to Dulbecco's phosphate-buffered saline, then placed in an anaerobic chamber (Plas-Labs, Inc, Lansing MI) containing nitrogen (95%) and carbon dioxide (5%) for 15 min at 37 °C, and finally, the chamber was sealed and incubated for 90 min at 37 °C (hypoxic–ischemic condition). OGD was terminated by changing normal media, and cell cultures reintroduced to the regular CO2 incubator (normoxic condition) at 37 °C for 1 h, of which period represented a model of “reperfusion” (Kaneko et al., 2014). After reperfusion, wells were randomly assigned to one of the treatment conditions, namely standard medium, IL-2/IL-2R, Tregs, or IL-2/IL-2R-treated Tregs at a concentration of 40,000 cells per well for three more days (Fig. 1). For the co-culture, the targeted cells were suspended in the treatment condition using 8-well poly-l-lysine plates, with each treatment condition done in triplicates. The co-culture was created using a two-chamber system with the targeted cells in lower chamber and the Tregs in the upper chamber, allowing us to conduct accurate cell counts of the targeted cells without contamination from the Treg population.

### Tregs Isolation and Culture

T-regulatory cells (Tregs), a subtype of T-cell, possess immunosuppressive properties. Tregs express the biomarkers CD25 and CD4 and have been found to exist as a small subpopulation of BMSCs. BMSCs were thawed and run through the depletion column. Anti-CD4 and CD25 antibodies were used to label Tregs. The bound antibodies were then conjugated with magnetic microbeads (Miltenyi Biotec, Bergisch Gladbach, Germany). Magnetically labeled cells were isolated by passing the cell suspension through a column containing magnetic metal substrate. First, non-CD4 cells were depleted using a depletion column. Then, CD25-positive cells were isolated from the CD4-enriched solution, resulting in a CD4/CD25 cell population of Tregs. Tregs were isolated on the same day as the start of cell culture to avoid any freeze–thaw cycle-associated damage.

### IL-2/IL-2R Complex

Recombinant murine IL-2 protein and anti-mouse IL-2 mAbs (JES6 –1) were used to make the IL-2/IL-2R complex (a kind gift by Dr. Xiaoming Hu). IL-2 protein was mixed with anti-IL-2 at a 2:1 molar ratio (1 μg of recombinant murine IL-2 protein and 5 μg of anti-IL-2 mAbs per 1 mL of media) and incubated at 37 °C for 30 min. IL-2/IL-2R complex was added directly to the Tregs’ culture medium.

### Rat Primary Cortical Cell Culture

E18 primary rat cortical cells were used (Neuromics; PC35102), hereafter referred to as PCCs. The substrate was coated with 50 μg/ml poly D-lysine (0.15 ml/cm^2^; Sigma P63407). Cells were diluted with NbActiv1TM (0.2 ml/cm^2^) and grown in coating plates at 37 °C. Half of the cell culture medium was changed every 3 days until the cells were confluent.

### Human Oligodendrocyte Progenitor Cell Culture

Human oligodendrocyte progenitor cells (OPCs) were derived from NIH approved H9 embryonic stem cells were used (EMD Millipore; CS2044696). A total of 10,000 OPCs were suspended in 400 μL human OPC expansion complete media (human OPC basal medium, 2% neural supplement 1 [50X], 0.04% recombinant human bFGF, 0.04% OPC expansion supplement A [PDGF-AA], and 0.04% OPC expansion supplement B [NT3]; Millipore; SCM106) and grown in Matrigel-coated 24-well plates at 37 °C (BD; 356,234). Half of the cell culture medium was changed every 3 days until the cells were confluent.

### Human Neural Progenitor Cell Culture

ReNcell CX immortalized cell line (SCC007, Millipore Sigma) derived from the cortical region of human fetal brain tissue was cultured to commit into neural progenitor cells (NPCs) according to the supplier’s protocol. The cells were plated onto BD Matrigel-coated T25 cell culture flasks (BD Biosciences, San Jose, CA, USA) and maintained in DMEM/F12 (Life Technologies, Grand Island, NY, USA) media supplemented with 2 μg ml^−1^ heparin (StemCell Technologies, Vancouver, Canada), 2% (v/v) B27 neural supplement (Life Technologies, Grand Island, NY, USA), 20 μg ml^−1^ EGF (Sigma-Aldrich, St Louis, MO, USA), 20 μg ml^−1^ bFGF (Stemgent, Cambridge, MA, USA), and 1% (v/v) penicillin/streptomycin/amphotericin-b solution (Lonza, Hopkinton, MA, USA) in 5% CO2 incubator at 37 °C. Half of the cell culture medium was changed every 3 days until the cells were confluent.

### Cell Viability Assay

The colorimetric 3-(4,5-dimethylthiazol-2-yl)-2,5-diphenyltetrazolium bromide or MTT reduction assay was conducted by following the instructions for use of Promega Corporation products (Cell Titer 96, Non-Radioactive Cell Proliferation Assay, Promega Corporation, Madison, WI, USA). This method assessed mitochondrial activity and thus cell viability by measuring the ability of cultured cells to convert yellow MTT to purple formazan dye. The supernatant and the cells were separated from the mixed culture at the end of the 3-h exposure time. Approximately 100 μL DMEM without phenol red was added, then 20 μL of the dye solution was added to each well, and the mixture was incubated on the plate at 37 °C for 3 h in a humidified, 5% CO_2_ atmosphere. After incubation, 100 μL of the solubilization solution/stop mix was added to each well, and the plate was allowed to stand overnight in the humidified, 5% CO_2_ incubator at 37 °C. The absorbance was quantified spectrophotometrically at a wavelength of 570 nm and with a reference wavelength of 900 nm in the BioTek Synergy HT 96-well microplate reader (BioTek Instruments, Inc., Winooski, VT, USA).

### TNF-α Immunocytochemistry

Cells were fixed for 5–10 min in 4% paraformaldehyde, permeabilized with a 0.1% tween solution, and incubated with fluorescent antibodies with affinity for rabbit monoclonal TNF-α antibody (Abcam; ab252382) at the manufacturer’s recommended dilutions. Comparison of TNF-α-positive cell counts between each group was performed using a DAPI nuclear stain (Vector Laboratories, Burlingame, CA, USA). Images were captured (20x) in randomly selected areas and analysis (ImageJ, National Institutes of Health, Bethesda, MD, USA).

### Statistics

Significant variations between means among the multiple groups in this study were assessed by one-way ANOVA. If there was a significant difference seen among the groups, a post hoc Bonferroni test was utilized for pairwise comparisons between means; *p* ≤ 0.05 was considered significant. Data are presented as mean ± SEM from quintuplicates of each treatment condition.

## Results

### IL-2/IL-2R-Treated Tregs Protect PCCs Against OGD/R

**Fig. 2:**
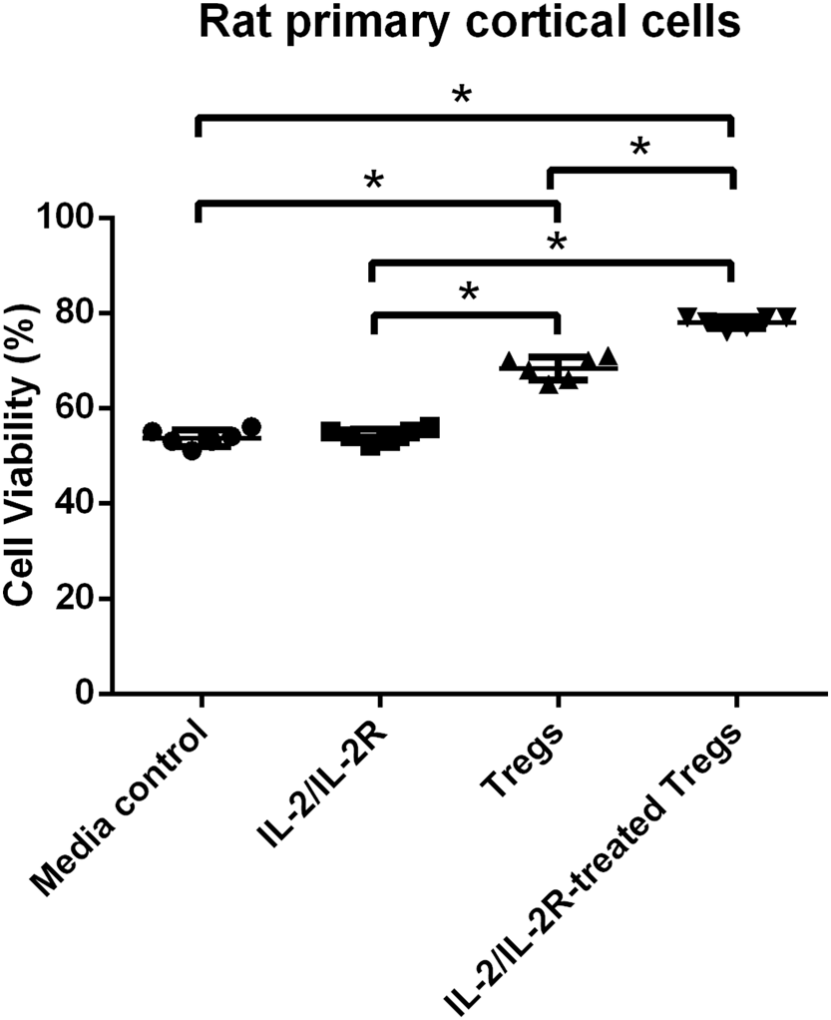
IL-2/IL-2R treatment facilitates Tregs neuroprotection of PCCs against OGD/R. OGD/R-exposed PCCs displayed significant cell death which was significantly reversed by both IL-2/IL-2R-Treg treatment and Treg treatment (*p < 0.0001), while cell viability with IL-2/IL-2R treatment alone was comparable to that of the media control treatment group (*p* > 0.05). Cell viability with the IL-2/IL-2R-Treg treatment was significantly higher than that of Treg treatment alone (*p < 0.0001)

The role of IL-2/IL-2R in facilitating Treg-induced neuroprotection was initially explored in OGD/R-exposed PCCs, which represented the cell type of the ischemic gray matter in the stroke brain (Fig. 2). One-way ANOVA analysis revealed a significant treatment effect (*F*_3, 20_ = 262.9). Pairwise comparisons revealed that OGD/R-exposed PCCs had a significantly improved cell survival in response to both IL-2/IL-2R-Treg treatment and Treg treatment (*p* < 0.0001), while cell viability with IL-2/IL-2R treatment alone was comparable to that of the media control treatment group (*p* > 0.05). Cell viability with the IL-2/IL-2R-Treg treatment was significantly higher than that of Treg treatment alone (*p* < 0.0001). These results indicate that IL-2/IL-2R-treated Tregs robustly increased cell viability of PRCs under stroke-like conditions.

### IL-2/IL-2R-Treated Tregs Shield OPCs from OGD/R

**Fig. 3:**
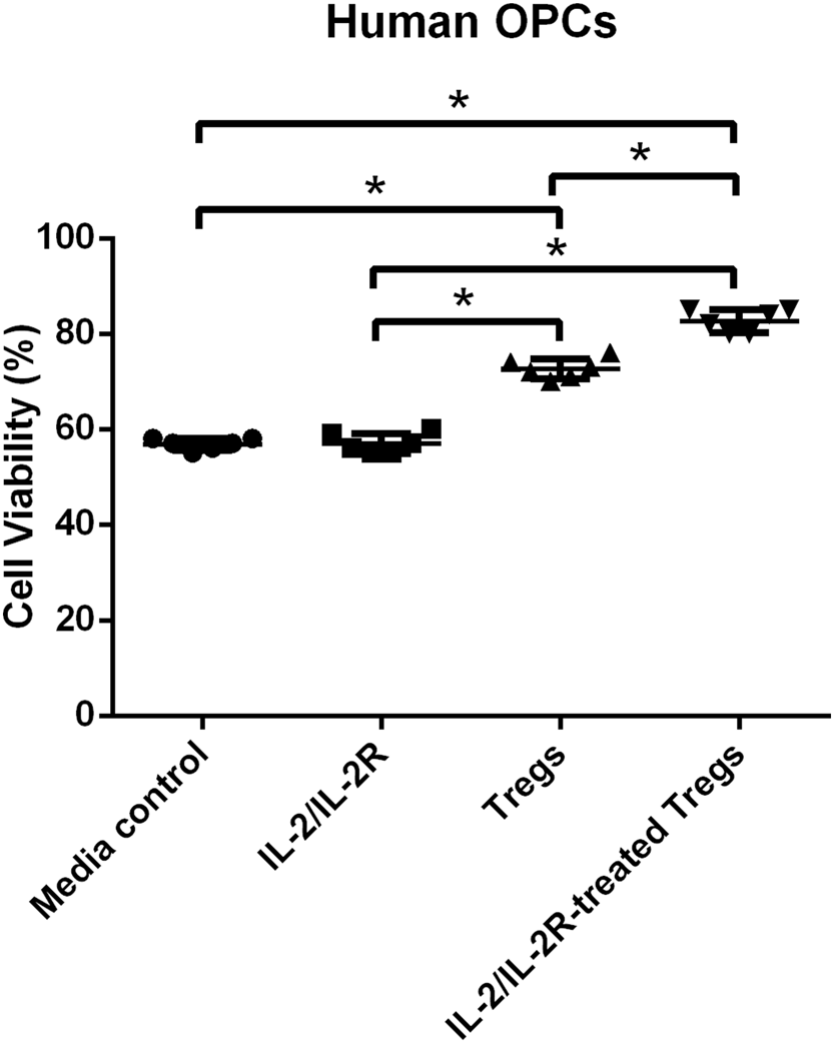
IL-2/IL-2R treatment enhances Tregs protection of OPCs from OGD/R. OGD/R triggered significant reduction in cell viability of OPCs but was significantly improved by IL-2/IL-2R-Treg or Treg treatment (*p < 0.0001), while IL-2/IL-2R treatment alone did not significantly improve cell survival, closely approximating that of the media control treatment group (*p* > 0.05). Cell viability with IL-2/IL-2R-Treg treatment was found to be significantly higher than with Treg treatment alone (*p < 0.0001)

The contribution of IL-2/IL-2R to Tregs’ neuroprotection was next explored in OGD/R-exposed OPCs, which corresponded to the cell type found within the ischemic white matter in the stroke brain (Fig. 3). One-way ANOVA analysis also showed a significant treatment effect (*F*_3, 20_ = 241.8). The post hoc Bonferroni test revealed that the OPCs displayed a significantly better cell survival when treated with IL-2/IL-2R-Treg or Treg treatment (*p* < 0.0001), while IL-2/IL-2R treatment alone did not significantly improve cell survival, closely approximating that of the media control treatment group (*p* > 0.05). Cell viability with IL-2/IL-2R-Treg treatment was found to be significantly higher than with Treg treatment alone (*p* < 0.0001). These results suggest that after OGD/R, OPC survival can be optimally improved by IL-2/IL-2R-treated Tregs.

### IL-2/IL-2R-Treated Tregs Attenuate OGD/R-Mediated Cell Death in NPCs

**Fig. 4:**
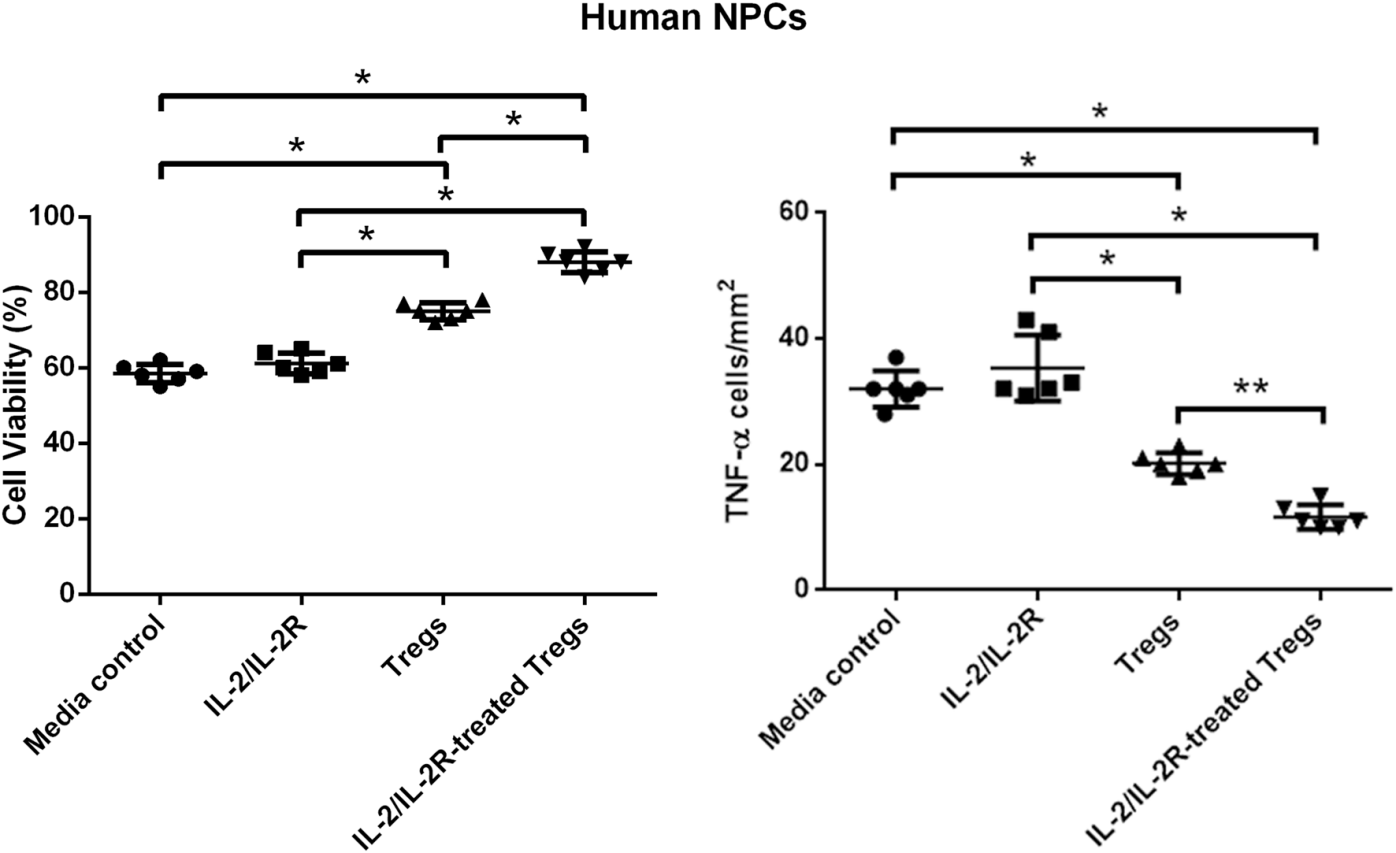
IL-2/IL-2R treatment increases Treg-induced survival of NPCs and reduction of inflammation. Left panel: OGD/R reduced cell viability of NPCs but was significantly blocked by IL-2/IL-2R-Treg treatment and Treg treatment (*p < 0.0001), while cell survival with IL-2/IL-2R treatment alone was comparable to that of the media control treatment group (*p* > 0.05). IL-2/IL-2R-Treg treatment was found to be significantly greater than with Treg treatment alone (*p < 0.0001). Right panel. OGD/R elevated TNF-α levels which was significantly suppressed by IL-2/IL-2R-Treg treatment (*p < 0.0001) or Treg treatment alone (*p < 0.0001), while IL-2/IL-2R treatment alone was ineffective in inhibiting TNF-α levels, comparable to that of the media control treatment group (*p* > 0.05). TNF-α levels with IL-2/IL-2R-Treg treatment were found to be significantly lower than Treg treatment alone (**p < 0.005). Under ambient condition (i.e., without OGD/R), only trace TNF-α levels were detected

Recognizing the multiple cell types in the neurovascular unit, we next tested the role of IL-2/IL-2R in OGD/R-exposed NPCs, which recapitulated both ischemic gray and white matter damage in the stroke brain (Fig. 4). One-way ANOVA revealed significant treatment effect (*F*_3, 20_ = 166.0). Pairwise comparisons showed that the NPCs had a significantly enhanced cell survival in response to both IL-2/IL-2R-Treg treatment and Treg treatment (*p* < 0.0001), while cell survival with IL-2/IL-2R treatment alone was significantly low comparable to that of the media control treatment group (*p* > 0.05). Similar to PCCs and OPCs, NPC viability with IL-2/IL-2R-Treg treatment was found to be significantly greater than with Treg treatment alone (*p* < 0.0001). These results indicate that IL-2/IL-2R-treated Tregs effectively protected NPCs against OGD/R-induced cell death.

### IL-2/IL-2R-Treated Tregs Dampen TNF-α-Associated Inflammation

In an effort to understand the mechanism of action, we then further assessed the production of the inflammatory marker TNF-α, which has been found to be upregulated after an ischemic injury, within NPCs after OGD/R (Fig. 4). One-way ANOVA demonstrated a significant treatment effect (*F*_3, 20_ = 66.84). Pairwise comparisons showed that the NPCs significantly suppressed TNF-α levels in response to both IL-2/IL-2R-Treg treatment and Treg treatment alone (*p* < 0.0001), while TNF-α levels with IL-2/IL-2R treatment alone was ineffective with upregulated TNF-α levels comparable to that of the media control treatment group (*p* > 0.05). TNF-α levels with IL-2/IL-2R-Treg treatment were found to be significantly lower than with Treg treatment alone (*p* < 0.005). These results demonstrate that the upregulation of TNF-α levels caused by OGD/R can be diminished by both Tregs, although more efficiently by IL-2/IL-2R-treated Tregs, indicating IL-2/IL-2R-Treg-mediated modulation of inflammatory response in stroke.

## Discussion

We provided evidence that IL-2/IL-2R-treated Tregs significantly mitigated cell death in both ischemic gray matter (PCCs) and ischemic white matter (OPCs) in vitro. The potential mechanism underlying IL-2/IL-2R-Treg-induced neuroprotection possibly involved modulation of inflammation, as evidenced by reduced TNF-α levels in cultured NPCs. Altogether, these data showed that IL-2/IL-2R-treated Tregs alleviated the injured neurovascular unit (NVU) following stroke by attenuating the inflammation-plagued secondary cell death.

The present study demonstrated the neuroprotective effects of the IL-2/ILR-Treg mechanism in OGD/R-treated cultured cells. Recently, intraperitoneal administration of IL-2/IL-2Ab enhanced Treg expansion in MCAO mice and substantially ameliorated motor impairment, diminished infarct size, and dampened inflammatory response (Zhang et al., 2018). Importantly, it was shown that IL-2/IL-2R treatment not only significantly increased the number of Tregs in mice, but also bolstered Treg function. Here, we observed that the IL-2/IL-2R-treated Tregs decreased the TNF-α frequency even more substantially than treatment with Tregs alone. IL-2/IL-2R administration alone did not significantly lower TNF-α concentrations. Taken together, the IL-2/IL-2R-Treg treatment afforded considerable cell survival effects against both white matter and gray matter ischemic injury in vitro. That Tregs can directly protect against ischemic neuronal injury entails Treg infiltration of the ischemic brain, which may occur in a relatively delayed manner, usually 5 days after stroke (Ito et al., 2019; Liesz et al., 2009). Therefore, the direct protective effect of Treg might not be timely to rescue neurons subjected to acute ischemic cell death. However, it is reported that neurons are highly immune regulatory and can govern the generation of Tregs in an inflamed brain (Liu et al., 2006), suggesting a potential cross talk between neurons and Tregs at subacute phase of stroke.

The NVU is responsible for directing the brain’s metabolic needs (Lo et al., 2009; Morrison et al., 2019). By maintaining the blood–brain barrier (BBB) and cerebral blood flow (CBF), the NVU serves a critical role in preserving homeostasis in the brain. The control and modulation of CBF depend upon neurovascular coupling. The NVU, composed of microvessels, astrocytes, neurons, axons, and their supporting cells, mediates the communication between neurons and their microvessels along with their intervening astrocytes (del Zoppo et al., 2010). Indeed, stroke damage to the NVU spurs cell dysfunction and cell loss. As CBF is cut off during an ischemic stroke, perturbed cellular electrical functions ensues leading to cell membrane failure and cell death due to the lack of energy reserves. Additionally, microvessel–neuron relationships suggest matrix response to focal ischemia. Following ischemia onset, neurons that were furthest from the microvessels were most likely to display injury. As focal ischemia limits the integrity of the matrix within the microvasculature and the loss of matrix-adhesion receptors, it leads to the rapid generation of matrix proteases in response to ischemia. Importantly, ischemia elicits responses within the microvasculature and nearby neurons to behave as a unit (del Zoppo et al., 2010). In the current study, we partially replicated the various components of the NVU by using PCCs, OPCs, and NPCs. Notably, we demonstrated that the damaged NVU can be ameliorated via administration of IL-2/ILR-treated Tregs.

In an effort to understand the mechanism of stem cell therapy in stroke, we recently demonstrated that Tregs, a subset of bone marrow-derived stem cells, improved stroke outcomes by reducing infarct volume and sensorimotor dysfunctions likely via an inflammatory signaling pathway (Zarriello et al., 2019). The primary function of Treg cells is to suppress the proliferation and function of other immune cells, especially effector T lymphocytes and to maintain immune homeostasis (Xia et al., 2016). The treatment of IL-2/IL-2R in mice significantly improves stroke outcomes, elevates Treg numbers, and increases the function and expression of CD39 and CD73 (Zhang et al., 2018). The modulation of post-stroke immune responses serves as an effective strategy to restrict ischemic brain injury and promote brain recovery. The IL-2/IL-2Ab stands as a clinically feasible immune therapy to bolster Treg responses and alleviate the effects of ischemic brain injury (Zhang et al., 2018). The current investigation has shown that Tregs confer neuroprotection in ischemic stroke. However, the role of IL-2/IL-2R in mediating Tregs is not well understood. Here, we observed that exposure to IL-2/IL-2R enhanced Treg-induced cell surviving effects, which correlated with attenuated TNF-α levels, indicating that IL-2/IL-2R facilitated the anti-inflammatory function of Tregs in sequestering cell death.

Mitigating the severity of secondary cell death responses to ischemic stroke is crucial to promoting brain rehabilitation. During an ischemic event, the loss of blood to brain tissue forms a hypoxic environment for neurons, endothelial cells, and many other cells that are vital to protecting the NVU (Liesz et al., 2015; Mehta et al., 2007). Stroke elicits necrosis and secondary cell death via proliferation of deleterious immune cells, proinflammatory cytokines, and complement immune systems (Chamorro et al., 2012; Liesz et al., 2015; Mehta et al., 2007). This extended inflammation has potentially damaging effects on the brain tissue and in some instances can result in further cell death (Jin et al., 2010). Remarkably, the current study demonstrated that IL-2/IL-2Ab treatment of Tregs suppressed the detrimental inflammatory response associated with cell death, supporting the application of Tregs in conjunction with IL- IL-2/IL-2R to curtail secondary cell death after stroke.

Pretreatment involving IL-2/IL-2R can potentially decrease the severity of an ischemic brain injury. IL-2/IL-2R treatment mitigates brain infarct and recovers motor functions after stroke (Zhang et al., 2018). IL-2/IL-2R treatment also decreased infiltration of inflammatory cells while also inhibiting the increase of inflammatory cytokines following an ischemic stroke. IL-2/IL-2R treatment combats ischemic brain injury by expanding Tregs in vivo and increasing the immunomodulatory function of Tregs (Zhang et al., 2018). These findings indicate that in vivo expansion of Tregs using IL-2/IL-2R may be a potential adjunctive treatment for stroke.

Stem cell transplantation in stroke reduces cell death in penumbra and peri-infarct areas via multiple regenerative mechanisms (Stonesifer et al., 2017; Zarriello et al., 2019). Tregs, a subpopulation of BMSCs, are able to augment the viability of cells through anti-inflammation in neurons that have been OGD/R (Neal et al., 2019). An increase in the concentration of Tregs resulted in a decline of the proinflammatory cytokine IL-6 after OGD/R (Neal et al., 2019; Zarriello et al., 2019). Tregs can alleviate inflammation following an ischemic event by guiding macrophage polarization to a higher regenerative M2 phenotype (Suenaga et al., 2015; Weirather et al., 2014; Zhou et al., 2017). A decline in Tregs concentration is observed following a stroke which could potentially serve a vital role in the aggravation of the inflammatory state that results in cell death (Dolati et al., 2018; Hu et al., 2014; Wang et al., 2018; Zarriello et al., 2019). Therefore, promoting Treg activity via the IL-2/IL-2R adjunctive treatment may substantially alleviate the severe inflammatory conditions that proceeds after ischemic stroke.

Although the IL-2/IL-2R-treated Tregs conferred significant cell survival effects, the present study did have some limitations. For instance, the targeted cell types, namely PCCs, OPCs, and NPCs, are immature with some stemness qualities thereby relatively resistant to OGD/R and not fully recapitulating the mature brain cells in the context of stroke. Interestingly, Tregs may be involved in neurogenesis, as Treg depletion inhibited neural stem cell proliferation 4 days after stroke (Wang et al., 2015). Moreover, Tregs may interact with other cells within the NVU, such as astrocytes (Ito et al., 2019), thus investigating other components of the NVU may reveal a better understanding of Treg regulation of the NVU and vice versa. In addition, our study focused on the role of IL-2/IL-2R in Treg-mediated neuroprotection. While IL-2 may represent a potent initial pathway candidate for Treg induction, there may be a multitude of other intricate signaling pathways that warrant further assessment. Lastly, the present study is in vitro and although there have been some in vivo studies examining the role of IL-2 in Treg-mediated neuroprotection in the gray matter (Hu et al., 2014), the efficacy of the IL-2/IL-2R-treated Tregs in mitigating white matter injury specifically is yet to be explored in stroke animal models. The direct effect of Tregs on OPC differentiation is reported in models of demyelination/remyelination (Dombrowski et al., 2017). The present observation that IL-2/IL-2R-treated Tregs enhanced OPC survival further supports the beneficial impact of this immune cell on white matter integrity. Another limitation is that we did not examine microglial and glial cell interactions with the neural cells. Because both microglia and glia have been recognized as not merely by-stander or supporting cells of the neurovascular unit, thus they participate closely in stroke pathology and treatment, an in-depth examination of their active roles in IL-2 and Tregs neurovascular protection is warranted. Finally, a time-dependent study is warranted in view of the temporal change in Treg numbers after stroke. In particular, an early depression followed by a quick recovery was observed in blood of stroke patients and in animal models. With the present study focused on CNS residential cells, it will be interesting to examine the time course of Treg infiltration, which starts at 5 days after stroke and escalates until at least 2 months after stroke (Ito et al., 2019). The eventual translation of the present in vitro study into an in vivo stroke model will further reveal the potentiation effects of IL-2 on Tregs neurovascular protection.

Overall, IL-2/IL-2R-treated Tregs exerted robust reduction of cell death in cultured cell models of ischemic white and gray matter more effectively than Tregs alone. The anti-inflammatory targeting of the TNF-α pathway may mediate the therapeutic effects of IL-2/IL-2R-treated Tregs. The present approach reveals a potential therapy to alleviate the secondary injury associated with stroke which currently manifests with limited treatment options.

## Data Availability

All data contained here are stored at the USF Center of Excellence for Aging and Brain Repair and available upon request.
